# Hydrogel‐Sheathed hiPSC‐Derived Heart Microtissue Enables Anchor‐Free Contractile Force Measurement

**DOI:** 10.1002/advs.202301831

**Published:** 2023-10-17

**Authors:** Yuta Kurashina, Keisuke Fukada, Shun Itai, Shuichi Akizuki, Ryo Sato, Akari Masuda, Hidenori Tani, Jun Fujita, Keiichi Fukuda, Shugo Tohyama, Hiroaki Onoe

**Affiliations:** ^1^ Department of Mechanical Engineering Faculty of Science and Technology Keio University 3‐14‐1 Hiyoshi, Kohoku‐ku Yokohama 223–8522 Japan; ^2^ Division of Advanced Mechanical Systems Engineering Institute of Engineering Tokyo University of Agriculture and Technology 2‐24‐16 Nakacho Koganei‐shi Tokyo 184–8588 Japan; ^3^ School of Integrated Design Engineering Graduate School of Science and Technology Keio University 3‐14‐1 Hiyoshi, Kohoku‐ku Yokohama 223–8522 Japan; ^4^ Division of Medical Science Graduate school of Biomedical Engineering Tohoku University 1‐1 Seiryomachi, Aoba‐ku Sendai Miyagi 980–8574 Japan; ^5^ Department of Mechanical and Systems Engineering, School of Engineering Chukyo University 101–2 Yagoto Honmachi, Showa‐ku Nagoya Aichi 466–8666 Japan; ^6^ Department of Cardiology Keio University School of Medicine 35 Shinanomachi Shinjuku‐ku Tokyo 160–8582 Japan; ^7^ Department of Pathology & Immunology Baylor College of Medicine One Baylor Plaza Houston TX 77030 USA

**Keywords:** anchor‐free, contractile force, engineered heart microtissue, hiPSC‐derived cardiomyocytes, hydrogel

## Abstract

In vitro reconstruction of highly mature engineered heart tissues (EHTs) is attempted for the selection of cardiotoxic drugs suitable for individual patients before administration. Mechanical contractile force generated in the EHTs is known to be a critical indicator for evaluating the EHT response. However, measuring contractile force requires anchoring the EHT in a tailored force‐sensing cell culture chamber, causing technical difficulties in the stable evaluation of contractile force in long‐term culture. This paper proposes a hydrogel‐sheathed human induced pluripotent stem cell (hiPSC)‐derived heart microtissue (H^3^M) that can provide an anchor‐free contractile force measurement platform in commonly used multi‐well plates. The contractile force associated with tissue formation and drug response is calculated by motion tracking and finite element analysis on the bending angle of the hydrogel sheath. From the experiment of the drug response, H^3^M is an excellent drug screening platform with high sensitivity and early testing capability compared to conventionally anchored EHT. This unique platform would be useful and versatile for regenerative therapy and drug discovery research in EHT.

## Graphical abstract

A hydrogel‐sheathed human induced pluripotent stem cell (hiPSC)‐derived heart microtissue (H^3^M) can provide an anchor‐free contractile force measurement platform in commonly used multi‐well plates. The contractile force associated with tissue maturation and drug response is calculated by motion tracking and finite element analysis on the bending angle of the hydrogel sheath.

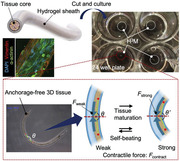

## 1 Introduction

In the development processes of living tissues from embryo to adult, mechanical forces generated in the maturating tissue are essential for emerging various characteristics of tissue morphologies and functions.^[^ ^1^ ^]^ Understanding the mechanism between those tissue maturation and mechanical forces has been one of the central topics in life science, especially in developmental biology. Recently, this mechanism has also become important from an engineering point of view due to the increasing demand for artificial tissue construction in the medical field, including regenerative therapy.^[^ ^2^ ^]^ For instance, artificially reconstructed engineered heart tissues (EHTs) promote maturity and maintain homeostasis by mechanical contractile forces generated by autonomous beating. With the technological advances in human induced pluripotent stem cell‐derived cardiomyocytes (hiPSC‐CMs),^[^ ^3^ ^]^ in vitro reconstruction of highly matured EHTs accelerated by contractile force has been attempted.^[^ ^4^ ^]^ These highly mature hiPSC‐derived EHT has been expected to mimic in vivo tissue kinetics for drug response, allowing the selection of cardiotoxic drugs suitable for individual patients before administration.^[^ ^5^ ^]^

For evaluating the function of the in vitro EHTs, mechanical contractile force generated in the EHTs during tissue formation and self‐beating motion is a critical indicator.^[^ ^6^ ^]^ The widely used method for measuring the contractile force of in vitro 3D EHTs is to attach the 3D tissue to a pillar‐like structure of known stiffness and to observe the deflection of the structure by the contractile force: the amount of the deflection is optically measured and converted into a contractile force by simple mechanics of materials. Contractile forces on the order of 0.1 mN have been measured for use in drug screening by placing elastomeric pillars on a culture substrate to hold the EHT,^[^ ^7^ ^]^ or suspending the EHT with two elastomeric wires.^[^ ^8^ ^]^ To observe the morphology of EHT at a higher dimension, the tissue response to drug testing is required in an in vivo‐like condition. That is, long‐term cultivation is desirable to promote tissue maturation.^[^ ^9^ ^]^ However, for a long‐time culture, there is difficulty to measure contractile force by anchoring EHT to the pillar because detachment and unstable shape retention of the EHT from the structure frequently occurs due to the increase in contractile force caused by the tissue formation and maturation. Furthermore, the pillar needs to be firmly covered by thick EHT (0.5–1.0 mm in thickness) for anchoring to prevent detachment. The thicker the tissue, the fewer nutrients diffuse into the tissue inside, resulting in the formation of a necrotic core.^[^ ^10^ ^]^

**Figure 1:**
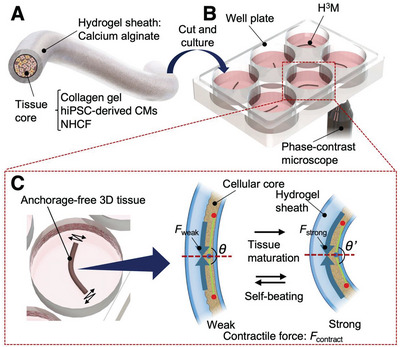
Platform of anchor‐free contractile force measurement by hydrogel‐sheathed hiPSC‐derived heart microtissues (H^3^M). A) A fiber‐shaped heart microtissues (HMTs) consisting of a hydrogel sheath composed of calcium alginate and a tissue core composed of human‐induced pluripotent stem cell‐derived cardiomyocytes (hiPSC‐CMs) and normal human ventricular cardiac fibroblasts (NHCFs) co‐cultured in a collagen gel was fabricated using a double coaxial flow microfluidic device. H^3^M was prepared by cutting fiber‐shaped HMT into 5 mm pieces. B) The H^3^Ms were cultured in a consumable well plate and observed with a phase‐contrast microscope. C) From tissue formation and self‐beating motion of the HMT in the H^3^M captured by a phase‐contrast microscopic camera, the bending angles (between *θ* and *θ*’) were measured. The contractile force, *F*_contract_, was calculated by finite element analysis from the bending angle (*θ* to *θ*’).

Here, we propose a platform for measuring the contractility of anchor‐free heart microtissues (HMTs) in a commonly‐used multi‐well plate by fabricating hydrogel‐sheathed hiPSC‐derived heart microtissues (HHHM: H^3^M, **Figure** **1**A,B). The contractile forces (CF) associated with tissue formation (formation‐based CF: FCF) and self‐beating (beating‐based CF: BCF) were calculated using motion tracking and finite element analysis of the deflection of the hydrogel sheath (Figure 1C). Due to the capability to measure contractile force by structural deformation of the hydrogel sheath, anchoring of the cellular tissue to the pillar‐like force‐sensing structure is no longer necessary. This means that our H^3^M can be cultured in consumable tissue‐culture wells and dishes without any additional force‐sensing systems. Furthermore, the hydrogel sheath keeps the 3D HMT in the fiber shape during long‐term culture, thereby avoiding necrosis by suppressing volumetric tissue growth. Therefore, rapid response to drugs can be evaluated stably even in a long‐term culture that is required for tissue maturation. In this paper, the FCF was evaluated when HMT was cultured for 7 days, and the BCF was analyzed along with heart beating rate before and after administrating heart drugs, isoproterenol, and propranolol, proving that our platform is capable of anchorage‐free measurement of the contractile force in micro‐sized in vitro EHT using consumable multi‐well plates (Movie S1, Supporting Information).

## 2 Results

### 2.1 Definition of Contractile Force Measurement using H^3^M

**Figure 2:**
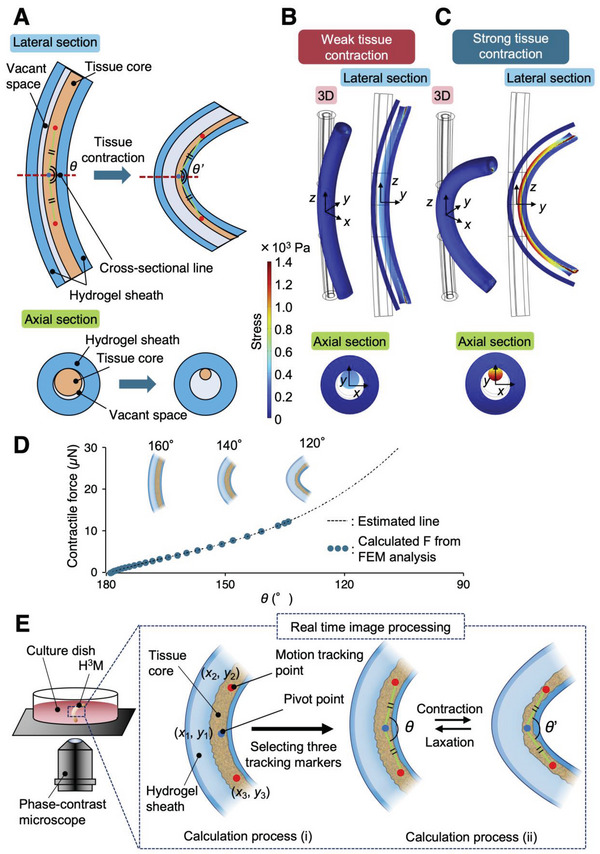
Angle referring force analysis (ARFA) using H^3^M. A) The compositional materials of the finite element method (FEM) simulation model of H^3^M from the lateral and axial cross section views. This model was constructed from a hollow hydrogel sheath and tissue core inscribe to the inner wall of the hydrogel sheath. The bending angle is defined by *θ* that is the vertex angle of an isosceles triangle with 350 µm sides formed at the center of the HMT. B,C) Through the FEM simulations, inner stress of the HMT (3D, lateral section and axial section), indicated by color scale, with B) weak and C) strong contractile forces was calculated. D) The FEM results was used to calculate *F* (third‐order polynomial approximation) induced in the tissue core when the H^3^M was bending at an bending angle, *θ*. An estimated line was calculated from a plot of 26 points (*R* ^2^ = 0.9996). E) The movement of the beating of the HMT in the H^3^M was captured by a phase‐contrast microscopic camera for motion tracking. E‐i) The coordinates (*x*, *y*, and *z*) of 3 tracking markers (pivot point (blue marker) and motion points (red makers)) were set for *θ* ∼ 160° by motion capturing. E‐ii) Real‐time calculation of the bending angle, *θ* on EHT was determined from the coordinates of the tracking markers.

Our H^3^M (Figure 1) consists of a 50–100 µm‐thick fiber‐shaped HMT covered with calcium alginate hydrogel sheath (outer thickness ≈200 µm) fabricated by a microfluidic device.^[^ ^11^ ^]^ In order to obtain the contractile force of the HMT during tissue formation or self‐beating motion, the relationship between the contractile force in the HMT and the bending angle of the H^3^M was calculated using a finite element method (FEM). Cross sectional schematic illustrations of the contracted HMT in the lateral and axial (red dashed line in the lateral view) directions are shown (**Figure** **2**A). The model of the H^3^M consists of a hydrogel sheath (100 and 200 µm in inner and outer diameters, respectively) on the outside and a tissue core (90 µm in diameter) on the inside. The increased contractile force as the HMT forms results in the hydrogel sheath beginning to bend. Moreover, the bending angle changes periodically due to contractile force from there during the self‐pulsation. The bending angle is defined by *θ* (Figure 2A), which is the vertex angle of an isosceles triangle with 350 µm sides formed at the center of the HMT. The tissue core is inscribed to the inner surface of the hydrogel sheath (Figure 2A) because the HMT in the core space is biased toward the bending direction of the H^3^M.

Based on the contraction behavior of the H^3^M above, an FEM model was constructed. The 3D view, lateral section and axial section of the FEM model of the EHT with the weak tissue contraction (Figure 2B) and the strong tissue contraction (Figure 2C) are shown. In this model, volumetric contraction of the tissue core causes bending of the outer hydrogel sheath because the core and the shell are constrained at the contact line. The color scale indicates the stresses that are caused by the contraction of the tissue core and the associated bending of the hydrogel sheath. The contractile force generated in the HMT can be defined as the integral of all stresses occurring in the normal direction (*z*‐direction) of the axial section (*x*‐*y* plane at *z* = 0) of the tissue core during tissue formation and self‐beating of the tissue core.

To estimate the contractile force of the H^3^M by the FEM model, the Young's moduli of the tissue core and the hydrogel sheath were defined as 1.7^[^ ^12^ ^]^ and 1.0 kPa,^[^ ^11a^ ^]^ respectively. By providing 0 to 81% volumetric contraction on the tissue core, the H^3^M model bent from 179 to 134°, and the calculated contractile force on the tissue core was 0 to 12.4 µN. From this FEM model, the relationship between the bending angle and the contractile force occurring in the H^3^M was calculated (Figure 2D, symbols indicate calculated points by the FEM model, dashed line indicates a third‐order approximation (*R* ^2^ = 0.9996)). Therefore, by using our proposed angle referring force analysis (ARFA) system, the bending angle of the H^3^M can be converted to a contractile force.

This ARFA calculates the contractile force from the optical image of the H^3^M, making it possible to calculate the contractile force in real‐time. This means that the change in contractile force during the beating of an HMT can be obtained by using a motion tracking algorithm on the images from the phase‐contrast microscope. For the beating motion of the H^3^M, the motion tracking markers are defined from the captured movies. The coordinates of the pivot point (blue marker (*x*_1_, *y*_1_) in Figure 2E) are selected at a certain point of the tissue core near the midpoint of the H^3^M. Then, two motion tracking points (red makers (*x*_2_, *y*_2_) and (*x*_3_, *y*_3_) in Figure 2E) are set in real‐time to form an isosceles triangle with *θ* at its apical angle to be ≈160°. This real‐time acquisition of the contractile force can be applied to the on‐demand evaluation of the HMT response to the drug administration.

### 2.2 Measurement of a Contractile Force of HMT Over Time

**Figure 3:**
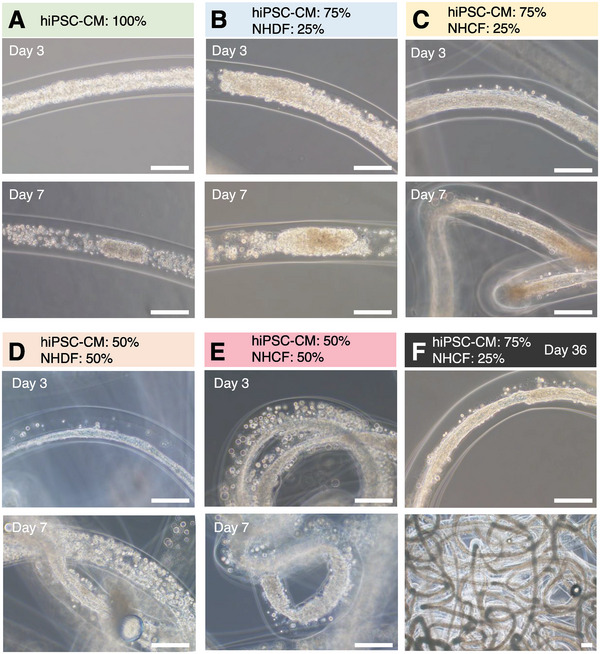
The fiber‐shaped HMT formed by co‐culture of hiPSC‐CMs and two types of fibroblasts. A–E) The fiber‐shaped HMT cultured in MEM α medium on day 0, 3, and 7 was observed by the phase‐contrast microscope. The ratio of co‐culture hiPSC‐CMs to fibroblasts was A) 100:0, B,C) 75:25, and D,F) 50:50, respectively. For the type of fibroblasts, normal human dermal fibroblasts (NHDFs) and normal human ventricular cardiac fibroblasts (NHCFs) were selected B,C) and D,E), receptively. F) Fiber‐shaped HMT was cultured for 36 days in FGM‐3 medium at 75:25 CM and NHCF, the optimal culture conditions for fiber‐shaped HMT. All scale bars = 200 µm.

The analysis of the contractile force was used for monitoring the generated force in the in vitro fiber‐shaped HMTs during culturing. The tissue cores were prepared by suspending 2.0 × 10^8^ cells mL^−1^ in 4 mg mL^−1^ collagen solution, and 1.5% sodium alginate was used as the sheath material. The fiber‐shaped HMT was prepared by gelation of the alginate sheath with calcium chloride. In order to optimize the stable culture conditions of fiber‐shaped HMT for H^3^M, hiPSC‐CMs were co‐cultured in MEMα medium with fibroblasts to mimic the in vivo condition of CMs. HiPSC‐CMs were mixed with normal human dermal fibroblasts (NHDFs) and normal human ventricular cardiac fibroblasts (NHCFs) in various ratios (**Figure** **3**A–E). Based on the evaluation of the culture medium in Note S1 (Supporting Information), FGM‐3 medium was selected. The fiber‐shaped HMT with a 75:25 of hiPSC‐CM and NHCF in FGM‐3 medium succeeded in obtaining optimal culture conditions, in which the beating was still visible after 36 days of long‐term incubation (Figure 3F; and Movie S2, Supporting Information).

**Figure 4:**
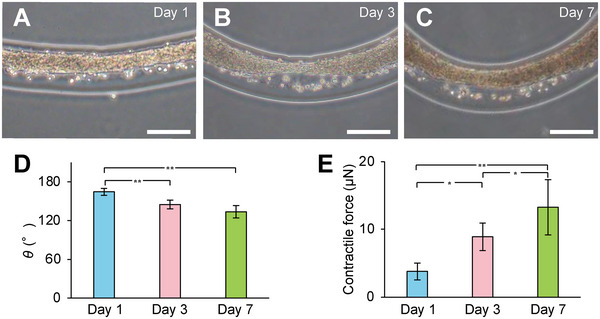
Measurement of the formation‐based contractile force (FCF) of HMT using H^3^M during culture. A–C) The H^3^M cultured from day A) 1, B) 3, and C) 7 were observed by phase‐contrast microscope. D) The bending angles were measured from the phase‐contrast microscopic images. E) The FCF at each culture time was calculated (mean ± S.D., *n* = 5, ^*^ *p* < 0.05, ^**^ *p* < 0.01, Student‐*t* test). All scale bars = 200 µm.

The analysis of the contractile force was used for monitoring the generated force during culture of the in vitro fiber‐shaped HMTs cut into 5 mm lengths, i.e., H^3^M. The H^3^M with a core diameter of ≈100 µm and a sheath diameter of ≈200 µm were fabricated. The H^3^M cultured in FGM‐3 medium was observed by phase‐contrast microscopy on day 1, 3, and 7 of culture (**Figure** **4**A–C). First, the cells were distributed on the collagen matrix within the alginate sheath with visible cell‐to‐cell borders on day 1 (Figure 4A). However, the cells adhered to each other to form an H^3^M on day 3 (Figure 4B). Moreover, the beating of the HMTs was observed on day 3. The H^3^M kept stable its tissue morphology with self‐beating on day 7 (Figure 4C). The bending angle (Figure 4D) of the H^3^M increased with the number of days of cell culture. The generated contractile force was estimated from the bending angle using the above ARFA of FEM analysis. The results (Figure 4E) indicate that the contractile force increased significantly as the day progressed. The contractile force of the HMT on day 7 increased up to 3.5 times that of day 1.

**Figure 5:**
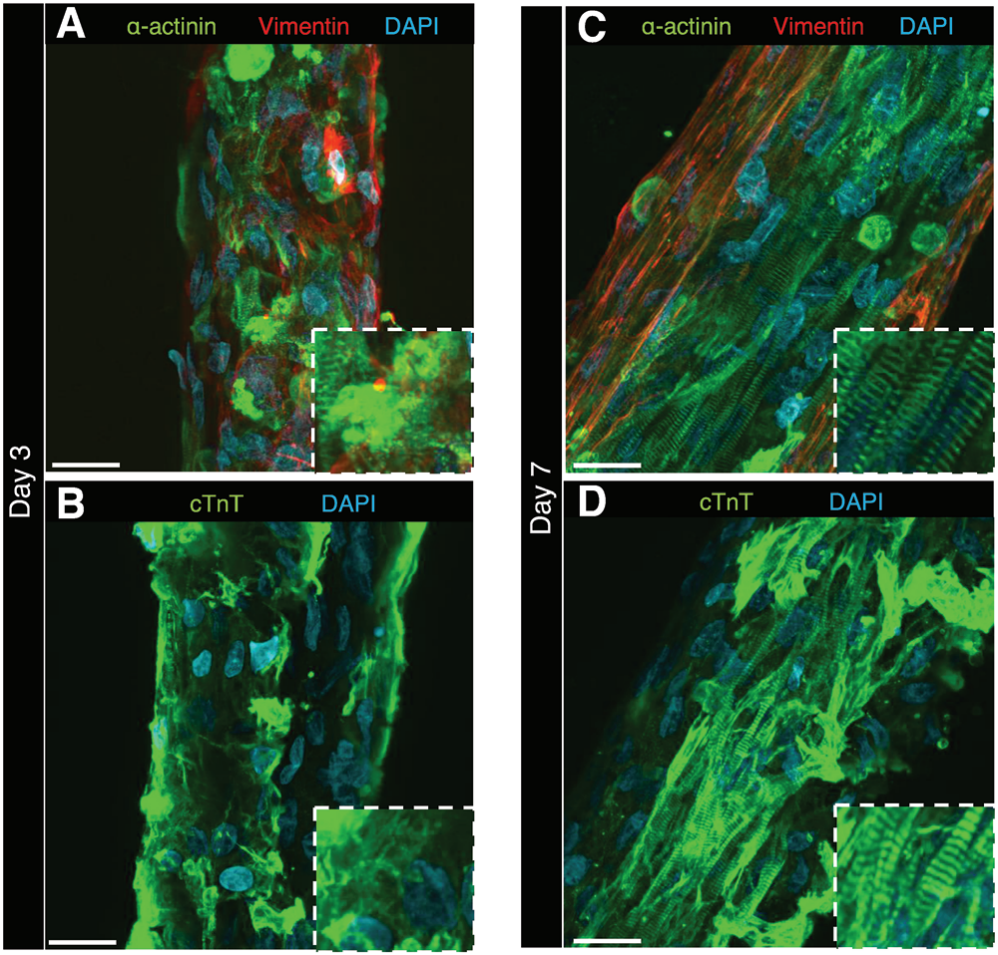
Confocal observation of the immunostained HMT. The HMT of day A,B) 3 and C,D) 7 days were immunostained. The insets in C) and D) show the magnified image of the sarcomere structures (× 2.5 times). A,C) α‐actinin, Vimentin and nuclei were stained green, red and blue, respectively. B,D) Cardiac muscle troponin T (cTnT) and nuclei were stained green and blue, respectively. All scale bars = 100 µm.

To understand this increase in the contractile force biologically, the immunostained HMTs were observed (**Figure** **5**; Figure S3, Supporting Information). Comparison of the immunostained images of α‐actinin and cardiac muscle troponin T (cTnT) between day 3 (Figure 5A,B) and day 7 (Figure 5C,D) using the confocal microscopy showed that the structure of sarcomeres was not observed on day 3, whereas that was clearly observed on day 7. The sarcomere, which is the smallest unit for generating the contractile force in muscle tissue, is the source of the contractile force in HMT. This means that the increase in the contractile force from day 3 to day 7 (Figure 4E) was associated with the expression of the sarcomeres of α‐actinin (Figure 5C) and cTnT (Figure 5D) significantly expressed on day 7, supporting the increase in contractile force based on the biological characteristics.

Interestingly, immunostaining of vimentin (fibroblast specific protein) analysis shows that the CMs were covered with NHCFs in the constructed HMT on day 7 (Figure 5C). This suggests that the CMs were maintained organized in the center of the HMT. This structure was so stable that the EHT was maintained and observed to beat even after 36 days. The cell density was maintained in this long‐term culture because the cell nuclei were in the center of the HMT (Figure S4, Supporting Information). This indicates that the measurement of the contractile force on the long‐term culture by ARFA is possible without the formation of a necrotic core.

### 2.3 Evaluation of Self‐Beating by Motion Tracking of the H^3^M

ARFA system can measure not only FCF associated with the tissue formation process in long‐term stable tissue culture, but also BCF due to self‐beating in general tissue‐culture wells and dishes without any additional pillars for anchoring tissues. This characteristic can be effectively employed to evaluate the efficacy on newly developed drugs for the treatment of EHTs.

**Figure 6:**
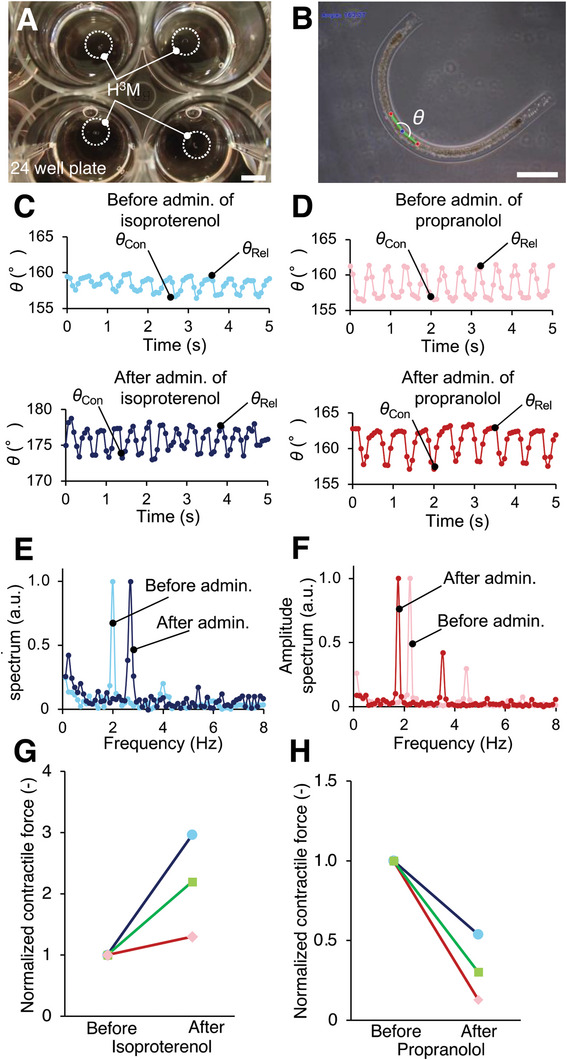
Measurement of the beating‐based contractile force (BFC) of HMT using H^3^M. A) As a demonstration for drug screening of engineered heart tissue (EHT), the H^3^Ms were cultured on a 24 well plate. B) Measurement of the bending angle, *θ*, by motion tracking based on pivot points and motion points. These 3 tracking markers were detected by the motion tracking algorithm of Kanade‐Lucas‐Tomasi tracker. C,D) The bending angle changes before and after administration of C) isoproterenol (a drug inducing positive inotropic effect) and D) propranolol (a drug inducing negative inotropic effect). The maximum and minimum bending angle, *θ*_Rel_ and *θ*_Con_ were calculated. E,F) The self‐beating rate change of the HMT before and after the administration of E) isoproterenol and F) propranolol was determined by FFT from the angle changes of C) and D), respectively. G,H) From the measured bending angles (A,B), the normalized BFCs before and after the administration of (C) isoproterenol and (D) propranolol were calculated. Scale bars of (A) = 5 mm and (B) = 500 µm.

To demonstrate the drug efficacy testing using the ARFA system, on‐demand measurements of self‐beating rate and contractile force were performed. Analysis of self‐beating was performed using H^3^Ms at day 7 (**Figure** **6**A). The movement of the beating of the HMT in the H^3^M was captured by a phase‐contrast microscopic camera for motion tracking (Figure 6B; and Movie S3, Supporting Information). As a demonstration of drug screening, changes in self‐beating rate were measured before and after administration of isoproterenol (a drug inducing positive inotropic effect) (Figure 6C) and propranolol (a drug inducing negative inotropic effect) (Figure 6D). The results showed that isoproterenol administration increased the self‐beating rate of HMT, and propranolol administration decreased the self‐beating rate of the HMT. The fast Fourier transform (FFT) analyses on the self‐beating motion before and after administration of isoproterenol (Figure 6E) and propranolol (Figure 6F) show that the self‐beating rate of the HMT increased from 2.0 to 2.7 kHz after the administration of isoproterenol and decreased from 2.2 to 1.8 kHz after the administration of propranolol, respectively. Therefore, the constructed H^3^M showed the ability to reflect the response of encapsulated HMT to cardiac disease drugs.

Furthermore, the contractile force, *F*_contract_, of the HMT (Table S1, Supporting Information) was calculated by the ARFA method from the bending angle, *θ*, extracted from the motion tracking. Analysis of the normalized BCF of the HMTs after isoproterenol (Figure 6G) and propranolol (Figure 6H) administrations shows that the contractile force increased in isoproterenol and decreased in propranolol as well as in the beating rate. Therefore, this platform of the H^3^M, in which the tissue core was encapsulated in the hydrogel sheath, enables the anchor‐free on‐demand measurement of the contractile force of HMTs.

### 2.4 Comparison of Drug Response to Conventionally Anchored EHT

**Figure 7:**
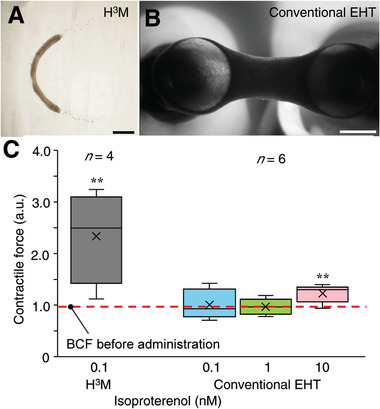
Comparison of H^3^M with conventional EHT in drug sensitivity. A,B) Evaluation of positive inotropic effect of isoproterenol using A) fiber‐shaped HMTs and B) conventional EHT. C) Drug testing with progressively increasing drug concentrations until significant differences occur (^**^ *p* < 0.01, *ANOVA*). Boxes have the meaning of 25% and 75% quartile around the population mean value (middle line = median) and error bars indicate maximum and minimum. Cross marks indicate the mean. Scale bars of A) = 500 µm and B) = 1 mm.

To compare the fiber‐shaped HMT fabricated in this study (**Figure** **7**A) with conventionally anchored EHT (Figure 7B), a controlled experiment of drug response was performed. Isoproterenol was administered to beat‐stable fiber‐shaped HMT (day 3) and conventional EHT (day 5). The concentration administered was increased from 0.1 nm until a significant difference in the contractile force was observed to evaluate the lower limit detection to isoproterenol (Figure 7C, absolute values are shown in Figure S4, Supporting Information). Note that constrictive force was measured 5 min after administration. H^3^M with the addition of 0.1 nm of isoproterenol caused a significant difference in the contractile force. Meanwhile, conventional EHT finally showed changes in the contractile force after dosing up to 10 mm. That is, the sensitivity of our fiber‐shaped HMT is ≈100 times higher than that of conventional EHT that was fabricated in the same fabrication conditions. Although large variability in the contractile force among fiber‐shaped HMTs compared to EHTs, notably H^3^M increased contractile force in all fiber‐shaped HMTs, as shown in the box‐and‐whisker plots. A high ratio of positive reactions should be a useful tool to prevent false positives and false negatives. In addition, our fiber‐shaped HMTs can be tested faster than conventional EHTs, requiring only 3 days of incubation before they start beating steadily for testing. Therefore, H^3^M is an excellent drug screening platform with high sensitivity and early testing capability.

## 3 Discussion

In this study, we proposed a platform for the anchor‐free measurement of the contractile force of the HMT with covered hydrogel shell, H^3^M. This platform frees us from the conventional method of anchoring macroscopic tissues to measurement devices required in the previous research.^[^ ^7^, ^8^ ^]^ For instance, conventional methods for measuring contractile force, such as cantilever^[^ ^13^ ^]^, microfluidic^[^ ^14^ ^]^, and 2D monolayer culture^[^ ^15^ ^]^ of cardiomyocytes, basically require anchoring cells to the culture dishes or the anchor points. This means that the evaluated tissues are difficult to remove from the culture dish, and the culture time is necessary for the cells to connect to each other for anchoring. Measurement of a cantilever^[^ ^16^ ^]^ can be performed directly by contacting the cellular tissue, but only localized contractile force of the tissue can be obtained. Throughput is also quite low due to the need to physically apply the cantilever. Meanwhile, our method does not require anchoring to a culture dish or anchoring points, making it easy to transfer the tissue to another dish in its tissue condition and eliminating the time required to incubate cells until anchoring. In addition, this method is very simple requiring only a video capture. Therefore, the measurement could be applied to a general multi‐well plate without the need to fabricate a complicated force‐sensing structure or to anchor the cultured macroscopic tissue to the force‐sensing structures. Hence, continuous visualization using ARFA system for the generated contractile force of the HMT was achieved with high affinity to the maturation of tissues for long‐term culture. By using this advantage of the ARFA system, the changes in contractile force caused by drug administration were also evaluated on‐demand. Smaller HMTs can be analyzed in the ARFA system than the EHTs in previous studies.^[^ ^17^ ^]^ The formation of H^3^M allows a sufficient supply of nutrients and oxygen to the cells in the center of the HMT, thus avoiding necrosis of the central cells. In addition, miniaturization of the EHTs can contribute to reduce the number of cells required to measure contractile force, saving the time and resources for tissue reconstruction.^[^ ^18^ ^]^ Certainly, conventional EHT can be used to build larger structures to fabricate stable and homogeneous microstructures. Meanwhile, fiber‐shaped HMTs have smaller numbers of cells due to the microscale of the tissue, which reflects the individual differences of the cells. In drug screening using tissues derived from iPSCs of patients, even the slightest differences between tissues are important because of the large individual differences in cells.^[^ ^19^ ^]^ Therefore, fiber‐shaped HMT has the advantage of high sensitivity and early testing because the number of cells used is small due to microscale tissue. Furthermore, considering the diffusion coefficient, small EHT is preferable for drug administration because the diffusion velocity of soluble drug is proportional to the square of the distance in the tissue. That is, when the radius of the EHT is 1/4 (example of this tissue: example conventional tissue^[^ ^20^ ^]^  = 100 µm: 400 µm), the cardiac drug will penetrate the EHT in 1/4^2^ of the time. Therefore, our H^3^M is highly effective for drug testing platform because the entire EHT can rapidly and accurately respond to the drug exposure.

Although the contractile force was measured during tissue formation and self‐beating, the measurement results of the contractile force on this platform show variations among individual H^3^Ms, especially in the evaluation of the drug administration. This is due to the difference in the initial bending angle of the formed H^3^M, caused by the difference in the generated contractile forces due to slight heterogeneity of the tissue core in the hydrogel sheath and individual differences in the CMs differentiated from hiPSCs. The contractile force in absolute values (BCF, Figure S4A, Supporting Information) and BCF/π*r* ^2^ (Figure S4B, Supporting Information), taking into account tissue heterogeneity, did not reach statistical significance, similar to conventional EHT (Figure S4C, Supporting Information) before and after drug administration. This is similar to studies showing the ratio of the contractile force in conventional 2D cardiac tissues^[^ ^21^ ^]^ and 3D EHTs^[^ ^21^, ^22^ ^]^ normalized by the value before drug administration. Therefore, comparisons between individual fibers in H^3^Ms may be difficult to make in experiments at different timed studies using different cells. However, this variation of the measured forces can be improved by massive and parallel analyses. A unique feature of the H^3^M is also the capability to form microtissue in the fiber shape, which is more than several meters in length.^[^ ^11^, ^23^ ^]^ By mechanizing the process to fabricate more uniformly and in larger quantities on the platform, up to thousands of microtissues are fabricated at a time by cutting the fiber‐shaped HMT into several millimeters in length as used in the measurement of contractile force. Thus, errors due to individual differences are reduced by using appropriate numbers of samples with multi‐well plates. The other feature of this platform is the ability to change the properties of the hydrogel sheath. By changing the thickness and material of the sheath, a weaker contraction force can be measured. Conventional measurement devices for contractile force use silicone‐based elastic materials such as dimethylpolysiloxane (PDMS). In our platform, alginate hydrogel ( = ≈1.0 kPa^[^ ^11a^ ^]^), which has a smaller Young's modulus than PDMS ( = 1.6–3 MPa^[^ ^24^ ^]^), can be applied to measure weak forces including those at the beginning of maturation.

Alginate, used in the sheaths of cellular tissues in this study, is a polysaccharide isolated mainly from brown algae. This is a linear copolymer composed of mannuronic acid (M) and guluronic acid (G), forming regions of M‐block, G‐block, and alternating structures (MG‐block). Because of the different structures of its mannuronic acid and guluronic acid, its physical properties vary according to their ratio (M/G). A high content of M residues leads to a flexible sheath, while a high content of G residues leads to a tough sheath.^[^ ^25^ ^]^ In addition, alginate hydrogels are cross‐linked with divalent cations including strontium and barium, not only calcium, in the gelation process. The physical properties of the obtained hydrogel change because these different divalent cations are selective in the cross‐linking blocks.^[^ ^26^ ^]^ Therefore, the properties of the sheath can be controlled by the M/G ratio and cross‐linking cations of the alginate. The physical properties can be optimized to match the required contractile force of the fiber‐shaped HMT. Cellular tissues using this alginate sheath have been fabricated in a variety of cell types. These include transplantation of encapsulated pancreatic islet cells into mouse,^[^ ^11^, ^27^ ^]^ formation of large liver‐like tissues^[^ ^11^, ^28^ ^]^ and osteoblast‐like tissues^[^ ^29^ ^]^ by assembling microfiber tissues, and mass culture of iPSCs in microfiber‐like tissues.^[^ ^30^ ^]^ Meanwhile, our study found conditions for long‐term culture of iPS cell‐derived cardiomyocyte tissue, which enabled measurement of contractile force. Based on our study, the tissue culture with alginate sheaths was extended to drug discovery research for cardiac diseases.

In our platform of H^3^M, drug diffusion occurs quickly without a necrotic core since our thin HMT consists of a small number of cells, and a large number of the HMTs can be arrayed and measured in parallel at a time. Because of these unique characteristics, this study shows drug‐inducing negative and positive inotropic effects. It is well known that hiPSC‐CMs or cardiac tissues exhibit immature profiles in morphology, sarcomere structure, electrophysiology and metabolism compared to adult cardiomyocytes, which are the main hurdles in drug discovery.^[^ ^4^, ^31^ ^]^ In regenerative therapy, transplanted immature hiPSC‐CMs induce post‐transplant ventricular arrhythmia.^[^ ^32^ ^]^ Fiber‐shaped HMTs have promise in providing mature EHTs that solve these problems. In addition, H^3^M can also be deployed as an attractive platform for the evaluation of cardiotoxicity of anticancer drugs.^[^ ^33^ ^]^ Due to the increasing incidence of chronic cancers of the heart, the importance of dealing with the cardiotoxicity of such cancer therapies has been intensified.^[^ ^34^ ^]^ For example, molecularly targeted drugs, including anthracyclines and small molecule kinase inhibitors, have been reported to have cardiotoxicity.^[^ ^35^ ^]^ Therefore, advanced prediction of cardiotoxicity is required for all upcoming anticancer drugs.

## 4 Conclusion

Our H^3^M provided an anchor‐free contractile force measurement platform in commonly used multi‐well plates. The contractile force associated with tissue formation and drug response was calculated by motion tracking and finite element analysis on the bending angle of the hydrogel sheath. This H^3^M has the potential to be an attractive platform with high sensitivity and early testing capability for the development of effective drugs by predicting drug‐induced cardiotoxicity in vitro in advance. From above, the H^3^M has a wide range of applications and is expected to be an innovative platform for the research of EHTs.

## 5 Experimental Section

### Double Coaxial Flow Microfluidic Device

To fabricate the fiber‐shaped HMT, a double coaxial microfluidic device based on a previously published work ^[^ ^11^ ^]^ was employed. This device was composed of glass capillaries and connectors made of resin. A glass capillary tube (outer diameter: 1.0 mm, inner diameter: 0.6 mm, G‐1, Narishige) was sharpened using a tip‐puller (P‐10, Narishige) and cut using a micro forge (EG‐44, Narishige). The tip diameter was adjusted to ≈200 µm. A square glass tube (outer diameter: 1.4 mm, inner diameter: 1.0 mm, 8100–100, VitroCom) was used to fix the inner glass capillary tube. A connector of these glass tubes was fabricated using a 3D printer (AGILISTA, Keyence). Those glass capillaries and connectors were assembled on a microscope glass slide (S2124, Matsunami Glass Ind., Ltd.). All inlets were connected to syringes via a three‐way stopcock (2‐9976‐01, As One) through Teflon tubes (JR‐T‐082‐M10, Shimadzu Corp.). All syringes were connected to syringe pumps.

### Cell Culture

HiPSCs obtained from Kyoto University (253G4, Kyoto University) were expanded on Matrigel‐coated dishes using hiPSCs expansion culture medium, StemFit AS103C (Ajinomoto).^[^ ^36^ ^]^ For the formation of the HMT, two types of cells were used. One was i) hiPSC‐CMs differentiated from hiPSCs obtained from Kyoto University (253G4, Kyoto University) for constructing the basic structure of the HMT. The hiPSCs were differentiated into CMs according to the conventional procedure.^[^ ^3b^ ^]^ Then, differentiated CMs were metabolically selected using glucose‐ and glutamine‐depleted with lactate medium, StemFit AS501 (Ajinomoto).^[^ ^3^, ^37^, ^38^ ^]^ The hiPSC‐CMs were cryopreserved on day 17 after differentiation, and the thawed CMs were employed for formation of the HMT. The other was ii) fibroblasts (passage 5–7). Two types of fibroblasts—normal human ventricular cardiac fibroblasts (NHCFs) and normal human dermal fibroblasts (NHDFs) were purchased from Lonza Walkersville, Inc. (NHCF, NHDF) and used for connection between hiPSC‐CMs to stabilize the HMT. All cells were maintained at 37 °C and 5% CO_2_ in humidified conditions.

### Formation of the Fiber‐Shaped HMT

A triple concentric laminar flow composed of core, sheath, and gelation flows was created in the microfluidic device for the formation of the fiber‐shaped HMT. For the core flow, a cell suspension containing hiPSC‐CMs and fibroblasts (totally 2.0 × 10^8^ cells mL^−1^) in type‐I collagen (4 mg ml^−1^, derived from the bovine dermis) (IAC‐50, KOKEN Co. LTD.) was prepared. For the sheath flow, 1.5% (w/w) sodium alginate solution (194–13321, Wako) dissolved in 145 mm sodium chloride (191–01665, Wako) was prepared and sterilized with a 0.22 µm filter. For the gelation flow, 100 mm calcium chloride (090–00475, Wako) solution was prepared and sterilized using an autoclave. Formation of the fiber‐shaped HMT was carried out at 4 °C to prevent the gelation of the collagen pre‐gel solution. For sanitizing, the microfluidic device was filled with 70% (v/v) ethanol for 20 min, followed by rinsing of the device with phosphate‐buffered salts (PBS, 163–25265, Wako). Then, the following steps were performed. i) The separated syringes were filled with the cell suspension for the core flow, with the sodium alginate solution for the shell flow, and with the CaCl_2_ solution for the sheath flow. ii) The syringe pumps sequentially started to inject the core flow (flow rate Q_core_ = 25 µL min^−1^), the shell flow (flow rate Q_shell_ = 75 µl min^−1^), and the sheath flow (flow rate Q_sheath_ = 2500 µL min^−1^). Since laminar flows were formed in the microfluidic device, each flow did not mix with the other. iii) The core flow was first filled with mineral oil using a three‐way stopcock, then switched to a cell suspension in collagen for the length of the formed the fiber‐shaped HMT to ≈1 m, and finally switched back to mineral oil to reduce cell loss. An H^3^M was formed in the microfluidic device and collected in a centrifuge tube filled with PBS. iv) The fiber‐shaped HMT in the centrifuge tube was transferred to a 100 mm culture dish. The PBS in the dish was replaced with the medium. MEMα (12 571 063, Gibco) with 5% fetal bovine serum (29‐172‐54, KAC) or FGM‐3 was employed with 1% penicillin‐streptomycin (P4458, SIGMA) as the medium for incubating the H^3^M. v) The H^3^M in the dish was incubated at 37 °C to solidify the collagen at the core. Subsequently, the fiber‐shaped HMT was cultured at 37 °C and 5% CO_2_ in humidified conditions. The fiber‐shaped HMT was cut into 5 mm pieces with scissors on day 1 of culture to prepare H^3^M.

### Immunofluorescent Staining

To visualize maturation of the hiPSC‐CMs and fibroblast co‐cultured tissue, the HMT was stained by immunofluorescent staining as follows. The HMT was fixed in 4% paraformaldehyde phosphate buffer solution (163–20145, Wako). After 15 min of fixation, the HMT was permeabilized with 0.1% Triton‐X100 (A16046, Alfa Aesar, MA, USA) in PBS for 10 min and soaked in 1% bovine serum albumin (BSA, A2153, Sigma‐Aldrich) in PBS to block nonspecific binding. Subsequently, the HMT was incubated with a primary antibody in PBS overnight. Next, the HMT was rinsed with PBS and incubated with a secondary antibody in PBS and DAPI (D1306, Invitrogen) for nucleus staining. After rinsing with PBS, the HMT was arranged on a 35‐mm glass base dish (3961‐035, IWAKI Co. LTD., Tokyo, Japan) and sealed with a mounting agent (Fluoromount/Plus, Diagnostic Biosystems, CA, USA). As primary antibodies, α‐actinin (A7811, Sigma), Vimentin (Ab92547, Abcam) and cTnT (Ab45932, Abcam) were used. α‐actinin was employed to observe cytoskeleton and intercellular adhesion, respectively, and both cells were stained. Vimentin was stained as an intermediate filament expressed only in fibroblasts. cTnT was a cardiac‐specific protein forming a sarcomere structure upon maturation.

### Evaluation of Contractile Properties by Finite Element Method

The contractile force of the H^3^M was calculated by the finite‐element method (FEM) analysis software (COMSOL Multiphysics®, COMSOL Inc.). The initial states of the iCHMS model were set as follows: the length of the fiber was over 2 mm, the diameter of the tissue core was 90 µm, and the inner and outer diameter of the hydrogel sheath were 100 and 200 µm, respectively. Note that the 2 mm length of the model was long enough for the calculation because the H^3^M had uniformity in the length. The tissue core and the hydrogel sheath were attached with one line at the inner edge of the core. Young's moduli of the tissue core and the hydrogel sheath were defined as 1.7^[^ ^12^ ^]^ and 1.0 kPa,^[^ ^11a^ ^]^ respectively. The tetrahedron meshes were formed on the model at the fine scale, which had 27 358 elements with side lengths of 24 to 196 µm.

For the boundary conditions, the center of the inner edge of the core was fixed at the point, and the rest edges of the center of the core was fixed at *y* = 0 for *y*‐axis. Also, the inner and outer edges of core and sheath were fixed at *z* = 0 for *z*‐axis.

By contracting the tissue core of the model at various ratio, the relationship between the bending angle and the contractile force occurring in the H^3^M was analyzed. The bending angle was defined as the vertex angle of an isosceles triangle with 350 µm sides formed on the inner edge of the core at the center of the model. To measure the angle after deformation, the displacement of each point of the triangle was extracted and calculated with the inverse function of the tangent. Besides, the contractile force generated in the H^3^M was calculated by integrating all stresses on the longitudinal cross sectional plane. From these values, the approximate curve in a third‐order approximation was generated between the bending angle and the contractile force.

### Motion Capture of the H^3^M

For the motion capture of the H^3^M, a computer vision algorithm was used. Specifically, the Kanade‐Lucas‐Tomasi (KLT) tracker,^[^ ^39^ ^]^ a widely used algorithm for image feature extraction and tracking, tracks the position of markers detected from the first frame of the video through the final frame. The KLT tracker was implemented using openCV‐python 4.5.2, a python3 package.

Next, from the motion tracking markers, the markers were selected to be used for the calculation of contractile force. For this calculation, markers located in isosceles triangles were required. Markers were selected that consisted of triangles with the largest angle of 160 ± 5 degrees, and the difference in the length of the sides was within 5% from the tracked markers. Of these, only the starting pivot marker was selected manually.

Using the above method, It was able to automatically extract the motion tracking markers necessary for the calculation of tensile strength by simply selecting the pivot markers from the video of the H^3^M.

### Drug Reactivity Analysis

As a demonstration of cardiac drug screening, changes in beating rate before and after the administration of isoproterenol (I6504, Sigma) and propranolol (P0884, Sigma) were observed. Isoproterenol causing positive changes in heart rate and contractility is a cardiac drug used in the treatment of atrioventricular block and bradycardia as a β‐stimulator. Propranolol causing negative changes in heart rate and contractility was a cardiac drug used in the treatment of angina pectoris, arrhythmia, and migraine as a β‐blocker. In order to use the HMT with a normal heart rate, the initial beating frequency of 1.0–1.5 Hz was selected. Isoproterenol or propranolol was dissolved in dimethyl sulfoxide (DMSO) to prepare a 1 mm stock solution. Then, the response of the HMT to isoproterenol or propranolol was observed by adding 0.1% of the prepared stock solution to the culture medium ( = 1 µm).

### Comparison with Anchoring of the EHT to the Pillar‐Like Force‐Sensing Structure

To compare the H^3^M in this study with the conventional method of measuring contractile force by anchoring, an EHT anchored to a pillar‐like force‐sensing was fabricated. The same lot of hiPSC‐derived CMs as the H^3^M for comparison was thawed from cryopreservation and incubated at 37 °C and 5% CO_2_.

The anchored EHT were prepared with agarose casting molds and solid silicone racks (C0002 and C0001, respectively; EHT Technologies), as previously described.^[^ ^40^ ^]^ In brief, casting molds were generated with agarose (2% in D‐PBS) in 24‐well plates (Corning, New York, NY). After solidification, the silicone racks were placed in the plates. The cells (75% hiPS‐CMs and 25% NHCFs, final concentration; 1 × 10^7^ cells mL^−1^) were mixed with 100 µL mL^−1^ Matrigel (356 230, BD Bioscience), 5 mg mL^−1^ bovine fibrinogen (A1153, Sigma‐Aldrich), 0.1% Y‐27632, 2×DMEM and 10% FBS. For each EHT sample, 100 µL of the reconstitution mix with 3 µL thrombin was pipetted into the casting molds. After fibrin polymerization (37 °C, 2 h), the silicone racks with attached fibrin gels were transferred to new 24‐well plates and cultured for up to 5 days at 37 °C and 5% CO_2_.

### Statistical Analysis

Results were expressed as the mean ± standard deviation. Samples were analyzed using the Student's *t*‐test and *ANOVA*. A value of *p < 0.05 or **p < 0.01 was considered significant.

## Conflict of Interest

H.O. is a co‐founder and director of Cellfiber, Inc. H.O. owns equity in Cellfiber, Inc. S.T. is an advisor of Heartseed, Inc. K.F. is a co‐founder and CEO of Heartseed, Inc. J.F., K.F., and S.T. own equity in Heartseed Inc.

## Supporting information

Supporting Information

Supplemental Movie 1

Supplemental Movie 2

Supplemental Movie 3

Supplemental Movie 4

Supplemental Movie 5

## Data Availability

The data that support the findings of this study are available from the corresponding author upon reasonable request.
