# Supplementary material for: Hydrogel‐Sheathed hiPSC‐Derived Heart Microtissue Enables Anchor‐Free Contractile Force Measurement
Source: Adv Sci (Weinh). 2023 Oct 17;10(35):2301831. doi: 10.1002/advs.202301831 (PMC10724413; doi:10.1002/advs.202301831)
Supplement: Supplementary file 1 — Supporting Information [file ADVS-10-2301831-s002.pdf]

ADVANCED SCIENCE
Open Access

Supporting Information

for *Adv. Sci.*, DOI 10.1002/advs.202301831

Hydrogel-Sheathed hiPSC-Derived Heart Microtissue Enables Anchor-Free Contractile Force Measurement

Yuta Kurashina, Keisuke Fukada, Shun Itai, Shuichi Akizuki, Ryo Sato, Akari Masuda, Hidenori Tani, Jun Fujita, Keiichi Fukuda, Shugo Tohyama and Hiroaki Onoe**

Supporting Information

Hydrogel-sheathed hiPSC-derived heart microtissue enables anchor-free contractile force measurement

Yuta Kurashina[#], Keisuke Fukada[#], Shun Itai[#], Shuichi Akizuki, Ryo Sato, Akari Masuda, Hidenori Tani, Jun Fujita, Keiichi Fukuda, Shugo Tohyama[*], and Hiroaki Onoe[*]

This PDF file includes:

Supplementary Text
Figures S1 to S4
Tables S1
Movies S1 to S2

Other Supplementary Materials for this manuscript include the following:

Movies S1 to S2

S1. Evaluation of optimal culture conditions for fiber-shaped heart microtissue (HMT)

At first, stable culture conditions for fiber-shaped HMT were determined. EHT in the heart contains not only cardiomyocytes (CMs) but also fibroblasts to maintain the tissue structure. In order to maintain stable fiber-shaped HMT, the co-culture conditions of hiPSC-derived CMs (hiPSC-CMs) and fibroblasts were evaluated in MEM α medium. hiPSC-CMs were mixed with normal human dermal fibroblasts (NHDFs) and normal human ventricular cardiac fibroblasts (NHCFs) in the ratios of 100:0, 75:25, and 50:50, respectively. CMs with NHDFs and NHCFs were cultured in fibroblast growth medium-2 and fibroblast growth medium-3 (FGM-2 and FGM-3, Lonza) with the provided supplement, respectively. These fiber-shaped HMTs were cultured for 7 days (**Figure 3**A–E). With 100% CMs (**Figure 3**A) or 75% CMs and 25% NHDFs (**Figure 3**B), a part of the HMT in the hydrogel sheath was fragmented on day 7. This indicates that the HMT was fragmented inside the alginate shell due to shrinkage of the CMs in the hydrogel sheath caused by the high ratio of CMs and the compatibility with fibroblasts. This suggests that the core of collagen gel containing fibroblasts is strongly characterized by the elongated tissue caused by culture with the hydrogel sheath. Compared with these co-culture conditions, the HMT composed of 75% CMs and 25% NHCFs was formed without fragmentation or elongation inhibiting tissue formation, and maintained even after 7 days (**Figure 3**C). The difference between NHDFs and NHCFs in the same fibroblast is due to the morphology of the cells. NHDFs cells are spherical with low migratory ability while NHCFs are spindle with high migratory ability [39]. These characteristics suggest that the highly migratory spindle-shaped NHCFs were suitable for filling the spaces between CMs and stably constructing fiber-shaped HMT. Therefore, the ratio of CMs and fibroblasts was decided to be 75:25 as the co-culture condition for the fiber-shaped HMT, and NHCFs were chosen as the fibroblast.

In order to select a suitable medium for culturing fiber-shaped HMT, we compared fiber-shaped HMT cultured in MEM α medium as a standard medium for hiPSC-CMs, and FGM-3 medium as a standard medium for NHCFs. In addition to fiber-shaped HMT in MEM α medium (**Figure 3**A–E), the HMT composed of 75% CMs and 25% NHCFs was cultured in FGM-3 medium observed by phase-contrast microscopy from day 0 to day 7 of culture (**Figure S1**). On day 3, the HMT cultured in FGM-3 medium was also formed as well as that tissue cultured in MEM α medium. Moreover, the beating of the HMT was observed in both MEM α (**Movie S1**) and FGM-3 (**Movie S2**) medium on day 3. These results indicate that the

CMs and NHCFs composed of the fiber-shaped HMT were long-linked and synchronized in beating.

To compare changes in HMT in long-term culture, the fiber-shaped HMT were cultured for longer duration with FGM-3 or MEMα medium. The results showed that the disruption of the fiber-shaped HMT in MEMα medium was confirmed on day 11 (**Figure S2**). Meanwhile, the fiber-shaped HMT in FGM-3 medium was stable and beating was observed even after 36 days (**Movie 2**). The result may suggest that the fibroblast activated in FGM-3 medium could mainly contribute to strengthens cell-cell junctions of CMs for maintaining the shape of the tissue.

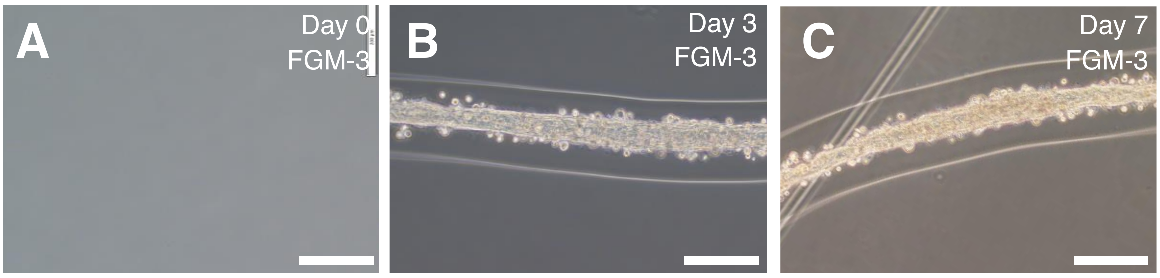

Supplementary Figure 1. The fiber-shaped heart microtissue (HMT) co-cultured with hiPSC-CMs and NHCF in various mediums. The ratio of hiPSC-CMs and NHCFs was decided to be 75:25 as the co-culture condition for the fiber-shaped HMT. The fiber-shaped HMT was observed on day (A) 0, (B) 3 and (C) 7. All scale bars = 200 μm.

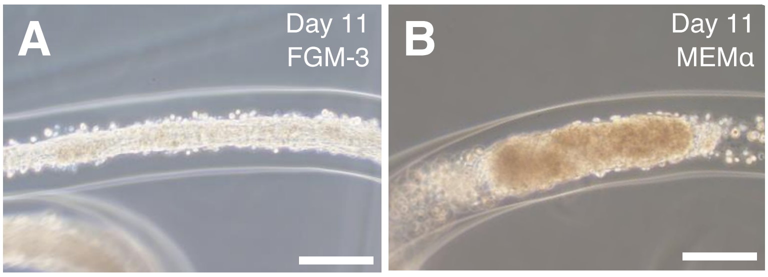

Supplementary Figure 2. The fiber-shaped HMT was observed on day 11. (A,B) The fiber-shaped HMT cultured in (A) FGM-3 and (B) MEM α were observed by phase-contrast microscopy. All scale bars = 200 μm.

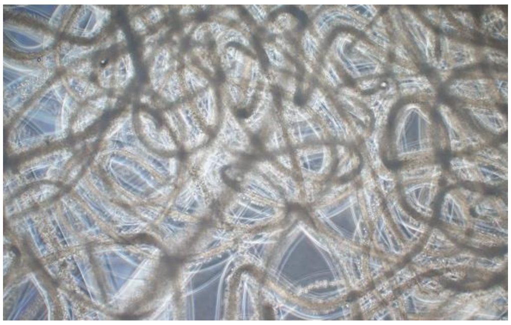

Movie S1 The beating of the HMT was observed in MEM α medium on day 3.

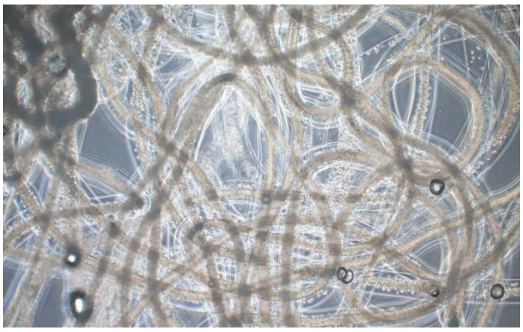

Movie S2 The beating of the HMT was observed in FGM-3 medium on day 3.

S2. Additional immunostained HMT

Confocal images of DAPI/α-actinin/Vimentin and DAPI/cardiac muscle troponin T (cTnT) on day 3 (**Figure S3**A and B) and day 7 (**Figure S3**C and D), respectively, as described in the results of the manuscript (**Figure 5**) are listed. In addition to these, confocal images of DAPI/α-actinin/Vimentin (**Figure S4**A) and DAPI/cTnT (**Figure S4**B) on day 36 are shown.

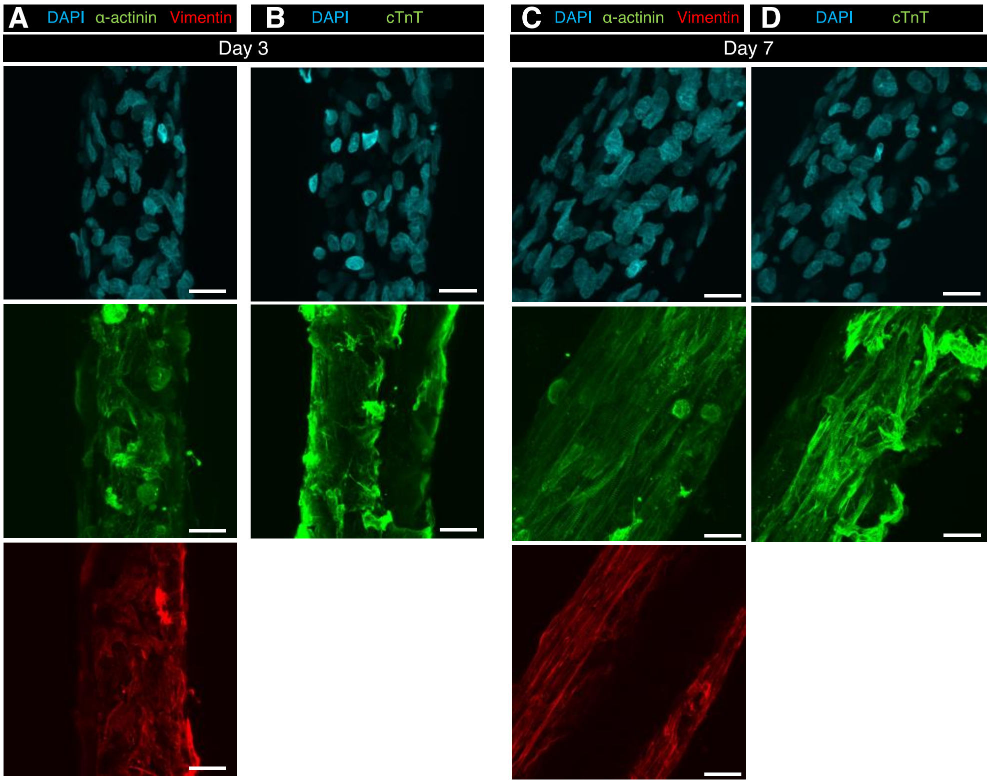

Supplementary Figure 3. Confocal observation of the immunostained HMT. (A) DAPI, α-actinin and Vimentin image on day 3. (B) DAPI and cTnT image on day 3. (C) DAPI, α-actinin and Vimentin image on day 7. (D) DAPI and cTnT image on day 7. All scale bars = 100 µm.

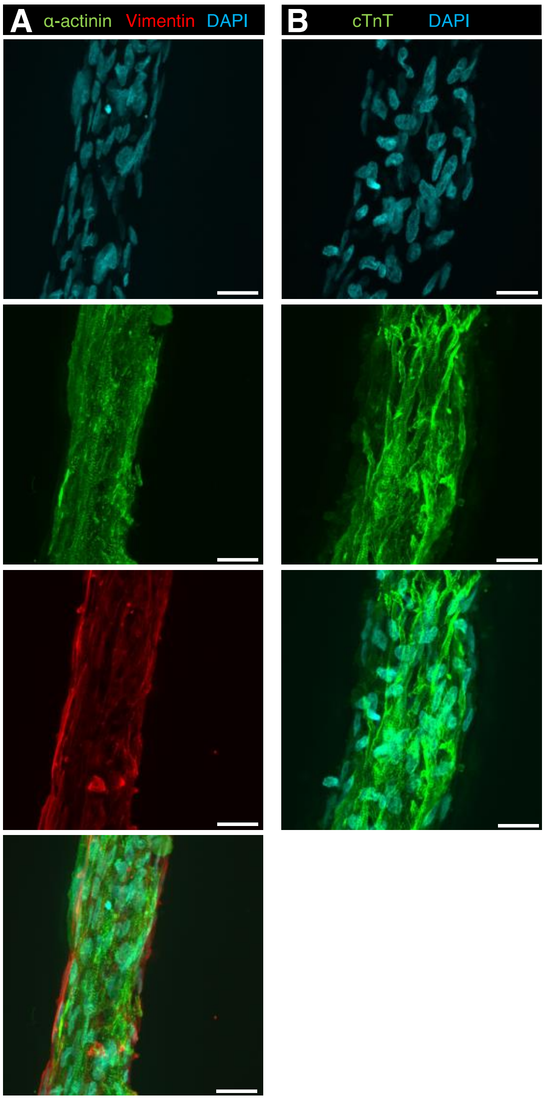

Supplementary Figure 4. Confocal observation of the immunostained HMT for 36 days. (A) DAPI, α-actinin, Vimentin and 3-color merged image. (B) DAPI, cTnT, 2-color merged image on day 36. All scale bars = 100 μm.

S3. Contractile force obtained by H^{3}M

Contractile force of HMT (**Figure 6**G,H) was measured by H^{3}M from the captured movies before and after the drug testing (Table S1). Relationship between the contractile force of fiber-shaped HMT before and after drug administration compared to conventional EHT (**Supplementary Figure 5**). Note that *r* represents as the radius of the fiber-shaped HMT at pivot point for calculation of bending angle.

Supplementary Table 1 Relationship between the bending angle and beating-based contractile force (BFC) before and after the drug testing for isoproterenol and propranolol.

Isoproterenol		Sample No.	1	2	3
Bending angle(°)	Max	Before	108.88	155.57	158.69
		After	89.93	158.84	155.81
	Min	Before	93.84	154.56	157.18
		After	64.38	156.56	153.92
Contractile force (μN)	Max	Before	27.94	5.88	5.15
		After	49.07	5.11	5.83
	Min	Before	43.87	6.12	5.50
		After	96.29	5.65	6.28
BCF (μN)	Max	Before	15.93	0.24	0.35
	Min	After	47.22	0.53	0.46

Propranolol		Sample No.	1	2	3
Bending angle(°)	Max	Before	146.19	105.17	145.95
		After	147.99	105.24	151.94
	Min	Before	138.11	86.49	144.02
		After	143.22	102.28	151.26
Contractile force (μN)	Max	Before	8.33	31.31	8.40
		After	7.82	31.25	6.78
	Min	Before	10.93	54.05	8.97
		After	9.22	34.18	6.95
BCF (μN)	Max	Before	2.60	22.74	0.58
	Min	After	1.40	2.93	0.17

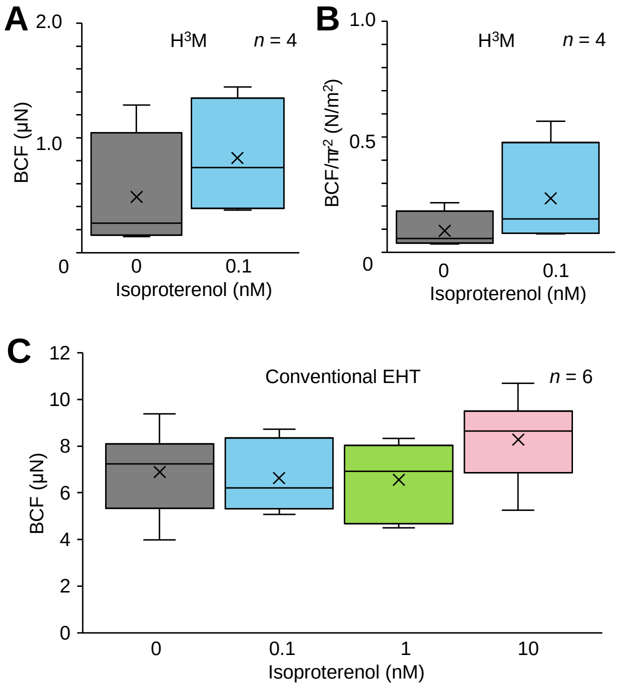

Supplementary Figure 5 Relationship between the contractile force before and after drug administration. (**A**) Absolute value of contractile force and (**B**) contractile force divided by cross-sectional area (πr^2) using fiber-shaped HMTs. (**C**) Absolute value of contractile force using conventional EHT. Boxes have the meaning of 25% and 75% quartile around the population mean value (middle line = median) and error bars indicate maximum and minimum. Cross marks indicate the mean.

Movie 1 Our platform for anchorage-free measurement of the contractile force in micro-sized *in vitro* EHT using consumable multi-well plates.

Movie 2 Macroscopic movie of the fiber-shaped HMT with a 75:25 of hiPSC-CM and NHCF in FGM-3 medium after 36 days of long-term incubation.

Movie 3 The measurement of the beating angle of the HMT in the H^3M was captured for motion tracking.
